# Costs of insecticide-treated bed net distribution systems in sub-Saharan Africa

**DOI:** 10.1186/s12936-020-03164-1

**Published:** 2020-03-04

**Authors:** Sara S. Scates, Timothy P. Finn, Janna Wisniewski, David Dadi, Renata Mandike, Mwinyi Khamis, George Greer, Naomi Serbantez, Sylvester Segbaya, Prince Owusu, Jules Mihigo, Lilia Gerberg, Angela Acosta, Hannah Koenker, Joshua Yukich

**Affiliations:** 1 PMI VectorWorks Project, Department of Tropical Medicine and the Center for Applied Malaria Research and Evaluation, Tulane University School of Public Health and Tropical Medicine, New Orleans, LA USA; 2 PMI VectorWorks Project, Johns Hopkins University Center for Communication Programs, Baltimore, MD USA; 3 Tanzania National Malaria Control Programme, Dodoma, Tanzania; 4 Zanzibar Malaria Elimination Programme, Zanzibar, Tanzania; 5 U.S. President’s Malaria Initiative, US Agency for International Development, Dar es Salaam, Tanzania; 6 U.S. President’s Malaria Initiative, US Agency for International Development, Bamako, Mali; 7 U.S President’s Malaria Initiative, U.S. Agency for International Development, Washington, D.C. USA

**Keywords:** Insecticide treated net, Continuous distribution, Mass campaign, Mass distribution, Universal coverage, Economic cost, Financial cost

## Abstract

**Background:**

Insecticide-treated nets (ITNs) are one of the most cost-effective measures for preventing malaria. The World Health Organization recommends both large-scale mass distribution campaigns and continuous distributions (CD) as part of a multifaceted strategy to achieve and sustain universal access to ITNs. A combination of these strategies has been effective for scaling up ITN access. For policy makers to make informed decisions on how to efficiently implement CD or combined strategies, information on the costs and cost-effectiveness of these delivery systems is necessary, but relatively few published studies of the cost continuous distribution systems exist.

**Methods:**

To address the gap in continuous distribution cost data, four types of delivery systems—CD through antenatal care services (ANC) and the expanded programme on immunization (EPI) (Ghana, Mali, and mainland Tanzania), CD through schools (Ghana and mainland Tanzania), and a combined community/health facility-based distribution (Zanzibar, Tanzania), as well as mass distributions (Mali)—were costed. Data on costs were collected retrospectively from financial and operational records, stakeholder interviews, and resource use surveys.

**Results:**

Overall, from a full provider perspective, mass distributions and continuous systems delivered ITNs at overlapping economic costs per net distributed (mass distributions: 4.37–4.61 USD, CD channels: 3.56–9.90 USD), with two of the school-based systems and the mass distributions at the lower end of this range. From the perspective of international donors, the costs of the CD systems were, for the most part, less costly than the mass distributions (mass distributions: 4.34–4.55 USD, Ghana and Tanzania 2017 school-based: 3.30–3.69 USD, health facility-based: 3.90–4.55 USD, combined community/health facility 4.55 USD). The 2015 school-based distribution (7.30 USD) and 2016 health facility-based distribution (6.52 USD) programmes in Tanzania were an exception. Mass distributions were more heavily financed by donors, while CD relied more extensively on domestic resource contributions.

**Conclusions:**

These results suggest that CD strategies can continue to deliver nets at a comparable cost to mass distributions, especially from the perspective of the donor.

## Background

Malaria continues to represent a significant public health challenge, despite being a preventable and treatable disease. Malaria is responsible for an estimated 216 million cases and 445,000 deaths globally, each year [1]. Children under 5 years of age and pregnant women are disproportionately affected by this disease. Malaria not only imposes considerable health impacts, but there is also evidence that it imposes substantial economic burdens on individuals, as well as entire economies [2, 3]. Since 2000, there have been increased funds and resources mobilized for the widespread control and elimination of malaria. As a result, there has been a rapid scale-up of existing effective anti-malaria interventions, particularly insecticide-treated nets (ITNs). This has led to unprecedented levels of vector control coverage across sub-Saharan Africa [4].

ITNs are the most widely used intervention for malaria control in Africa, representing the main vector control tool in nearly all malaria endemic African countries [1]. They are effective for reducing malaria-related morbidity and mortality by acting as a direct barrier to mosquito biting and by providing community-wide protection through killing of mosquitoes resulting in reductions in vector density and average lifespan [3]. The cost-effectiveness of ITNs in the prevention of malaria has been demonstrated in a variety of settings [2, 3, 5, 6]. While ITNs have been highly effective at reducing prevalence and incidence across the continent, sustaining and increasing access to these interventions remains a concern. Maintaining high ITN coverage is particularly problematic due to the continuous loss of nets from households due to wear and tear, repurposing, or movement of nets out of target areas [4].

In 41 of 45 countries in the World Health Organization (WHO) African Region, the policy is to distribute ITNs free of charge [1]. The WHO recommends that to achieve and maintain universal ITN coverage, countries should apply a combination of mass and continuous distributions through multiple channels, including ANC and EPI [7]. Mass distributions have been identified as an excellent tool for “catch-up”– if carried out efficiently and successfully they are able to rapidly and efficiently increase coverage and usage of nets on a large scale [8–22]. Equity (i.e. socio-economic disparities in access as measured by the equity ratio) has shown to be relatively high with this channel, as all households are targeted and there is little evidence that poorer households benefit less [19, 22–25]. Historically these distributions have been shown to be quite cost-effective [5, 8].

While mass distributions are a cost-effective way to quickly achieve high coverage over a particular area, coverage gaps begin to appear almost immediately post-distribution through net deterioration, loss of nets, and population growth, therefore, requiring complementary continuous distribution channels to sustain or “keep up” coverage over time [8, 26–28]. Models have suggested that relying solely on mass distributions without keeping up access through health facility-based systems—through ANC and EPI (sometimes also called “routine distribution”) would result in lower levels of access [29]. This effect is even more apparent if the time between mass distributions is assumed to be 5 rather than 3 years, underlining the need for continuous systems that provide constant access to ITNs [29]. While the WHO recommends that mass distributions be implemented every 3 years, often distributions are delayed so that the gap is longer than 3 years [7]. This recommendation is based on the assumption that the useful life of an ITN is 3 years. However, in reality, the lifespan of ITNs may actually be closer to two rather than 3 years [30–33]. Because of these gaps in coverage, there are still vulnerable households who need replacement ITNs between distributions. Ideally, households with worn-out nets would have the opportunity to replace them without waiting for another mass distribution; similarly, migrants new to an area and children born between mass distributions would also be able to obtain a net. Evidence also suggests that spikes in malaria cases may occur between mass distributions underscoring the necessity for additional ITN distribution between mass distributions [34].

Continuous distribution (CD) strategies employ channels other than mass distributions to deliver ITNs and comprise routine ITN delivery at ANC and EPI, schools, community-based (local political/community leaders), and sales within private sector, including social marketing. Despite the WHO recommendations for a combined approach utilizing both mass campaigns and CD systems to deliver nets, most countries are still relying heavily on mass distributions to distribute ITNs [35]. Data reported by NMCPs indicate that, between 2014 and 2016, mass distributions accounted for 75% of ITNs distributed in sub-Saharan Africa, while ANC accounted for 13% and EPI for 5% [1]. However, a 2016 study demonstrated that among 48 malaria-endemic countries in Africa, 33 malaria programmes had policies for ANC-based CD of LLINs, and 25 had policies for EPI-based CD [35]. ANC and EPI may be additionally advantageous as ITN distribution points because their target populations are biologically vulnerable to malaria. More countries are beginning to implement CD of nets through schools and communities (through local political/community leaders). School-based distributions have been evaluated in Ghana, Tanzania, and Nigeria [36–39] and community distribution programmes have been successfully piloted in a number of locations, including Madagascar, South Sudan, and Zanzibar [40, 41].

The existing knowledge base on the costs and cost-effectiveness of CD systems is sparse. Only a few studies were identified that describe the costs of health facility-based CD [5, 42, 43]. Those that do exist utilize different methodologies for estimating costs, limiting the ability to draw meaningful comparisons. Only one study was identified that estimated the cost of a community-based distribution programme [40]. In this study, only financial costs were included, and the methodology used to estimate costs differed significantly from that used to estimate the costs of health facility-based CD in the aforementioned studies. The financial cost for the community-based CD program was reported to be 19.21 USD (18.81 USD when deflated to 2017 USD) per net distributed. Studies estimating the cost of delivering nets through school-based distribution programs were not identified in the literature, though a small number of studies were identified that evaluated the effects of these systems on net ownership and access [36–39]. As countries continue to scale up combination delivery systems, they will require information on the costs of these various distribution channels, as well as information about how these costs relate to other outcomes, such as coverage and malaria incidence. This data will better inform policy decisions about when and under which conditions (financial and epidemiological) these systems should be implemented.

This manuscript describes a study of the costs of four types of delivery systems (mass distributions, CD through routine health services (ANC and EPI), schools, and a combined community/health-facility distribution) delivered at scale in various country contexts, utilizing a consistent method for cost collection and analysis for all programmes. The objective of this study is to compare the costs of the four delivery systems.

## Methods

### Programme selection

The costs of four types of delivery channels were analysed in this study. These delivery channels were classified as either CD or mass campaigns. Distributions implemented at intervals of 3 years or greater with the goal of achieving universal coverage were classified as mass campaigns while all other distributions were classified as CD. Intermittent approaches, like the school distributions analysed in this study, while not purely continuous strategies (as nets were only made available once per year to students in eligible classes), were classified as CD strategies. Like other CD strategies, school distributions aim to boost coverage in the interval between mass campaigns and rely heavily on lower government levels to function effectively. Three school-based CD programmes were included in this study: two in Tanzania (2015 and 2017) and another in Ghana in 2016. Four health-facility based CD programmes were included: two in Ghana (2015 and 2016), one in Mali (2015), and one in Tanzania (2016). One combined community/health facility-based CD programme was included (Zanzibar 2015). Two mass campaigns were also included (Bamako, Mali 2015 and Segou, Mali 2015). The ITN programmes chosen for this analysis were deliberately selected to represent different ITN programs and delivery channels in sub-Saharan Africa.

### Intervention description

A description of each intervention was developed based on document reviews (operational records and logistics documents from key stakeholders, donors, and partner organization), and through key informant interviews with individuals involved in the management, supervision, and logistics of the distribution programmes at all levels; this includes donors, partner organizations, members of the National Malaria Control Programmes (NMCPs), and individuals at the local health and school levels (districts, subdistricts, health facilities, schools, and local political/community leaders). The intervention description guided all further data collection components by outlining and delimiting the appropriate resource inputs to be costed and partner organizations which had to be identified and contacted.

### Types of costs included

Because the costs were analysed from the provider perspective, all costs associated with provision of the health care intervention are included from the perspective of the providers of the intervention (including NMCPs, health care workers, international donors, philanthropic organizations). Household costs, whether direct or indirect, were not included. All direct costs of the program to the providers were included, including commodities, social and behaviour change communication, transport, payment of salaries, and volunteer time. No cost-savings to the provider due to reduced treatment were included.

Additionally, costs were also analysed from the international donor perspective, meaning only the subset of costs incurred (financed) by international donors were included in these sub-analyses. Costs incurred (financed) by domestic government agencies (e.g. the NMCPs, and Ministries of Health) were not included in these sub-analyses.

### Data collection

Cost data were collected retrospectively from the financial and operational records kept by partner organizations and through interviews with programme managers and implementers involved in ITN distribution activities. Data were collected by examining the agencies’ financial records, including budgets, expenditure records, reports, receipts and invoices; as well as operational records, such as reports and published documents. The ingredients approach was used for each of the programs analysed, however for certain line items and programme activities, if the information was deemed too sensitive to be released as inputs and prices (i.e. national level personnel salaries), or was not available in adequate detail (i.e. costs only estimable from financial reports), aggregated expenditure was used. Aggregate costs were most commonly used for line items such as “indirect costs”, “other direct costs”, and national level salaries, and often components of training, planning, supervision, SBCC, equipment, storage, and transport costs at the national level. Aggregate costs were never used for nets and were rarely used for costs obtained at the lower levels (district and beyond), such as district or facility personnel and fringe costs, transportation costs, or storage costs.

When costs or resource inputs were not reflected in financial or other available records (*e.g.* government human resource costs) information was collected through stakeholder interviews or, in the case of the third round of the Tanzania School Net Program (SNP3) and Ghana analyses, a resource use survey instrument was utilized.

Resource use was valued at three levels: (1) national and international, (2) the district, subdistrict, or circuit (the subdistrict level for schools in Ghana) (3) and, peripherally, at health facilities or schools (or other distribution points). Resources were generally valued using opportunity costs derived based on the reported expenditures or budgets and, in the case of personnel, on salary plus fringe benefits. Capital goods were valued based on their procurement costs or, in the case of building rents, on the average market value of similar properties. Where data was unavailable, the World Health Organization-CHOosing Interventions that are Cost-Effective (WHO-CHOICE) database was used for valuation purposes, after conversion to non-purchasing power parity-adjusted United States Dollars (USD) [44].

Interviews were also conducted to collect resource use and expenditure information at lower governmental levels (districts/communes, health facilities, schools). Key individuals, such as members of the district health management teams, malaria focal persons, health facility staff and teachers responsible for distributing nets, and others that were involved in the management of the distribution programmes at the lower levels were interviewed. Data collected from these interviews were then used to make assumptions about estimated resource and time use, as well as actual expenditures and costs for specific distribution activities at lower health system levels. An average cost for all districts, all schools, and all health facilities interviewed or surveyed was calculated and then applied to all participating districts/schools/health facilities.

### Cost classification and adjustments

Costs were divided into capital and recurrent costs, based on the lifetime of the goods or service being purchased. Capital costs are costs incurred to purchase goods or services with a life span longer than 1 year. Recurrent costs are costs incurred for goods or services lasting less than 1 year. Capital costs were discounted in the economic analysis using lifetimes and discount rates determined through stakeholder interviews, expert information, and past literature. Varying discount rates and lifetimes were examined in sensitivity analysis. Both financial and economic analyses were conducted. These two types of analysis show (1) financial costs—what the actual expenses of running a programme were, and (2) economic costs—the value of all resource use during the study period. In the financial analysis, capital costs were not discounted and were instead applied in full at the time of the purchase.

The costs of the programmes included in this paper were collected in a range of international currencies over a period of time dating back to 2015. In order to draw meaningful comparisons between costs from different programmes, costs were adjusted to a common year and a single currency. Costs of interventions were first converted from local currency to US Dollars using the exchange rate at the year the initial costing analysis was performed. These costs were then split into tradable costs (e.g. nets) and non-tradable costs (e.g. personnel, training, supervision, SBCC). The estimated costs of tradable goods and services were inflated or deflated, in the case of the 2017 Tanzania School Net Programme, to 2016 USD using the consumer price index (CPI) adjustment [45]. The estimated costs of non-tradable goods and services were first inflated or deflated to 2016 USD, using the consumer price index (CPI) adjustment, and then converted into 2016 International Dollars (Int$) using the purchasing power parity ratios for the countries in which the costs were incurred [46]. The International Dollar (Geary–Khamis Dollar) is a hypothetical unit of currency that has the same purchasing power that the US dollar has in the USA at a given point in time [45]. The Int$ represents the amount of money that would be necessary to purchase an identical bundle of goods and services in the US as was actually purchased in the country of interest. When working in lower income countries (with lower price levels), this adjustment will increase the nominal total cost number compared to the USD total. For this reason, results are presented in both 2016 USD and in 2016 Int$ in order to give a more comparable number (Int$) and a value which is more interpretable to most readers (USD).

### Outputs

The primary output measure used in the analysis was the number of nets distributed. These data were collected from partner organization records. The number of nets distributed was used to calculate the cost per net distributed. A second combined output measure was also calculated—cost per person-year of protection (PYP), which assumes that, in the base case scenario, one net can cover two people for a period of 3 years. The primary output was also used to calculate a third combined output measure—cost per treated-net year (TNY), which assumes a lifetime of 3 years per net in base case scenario calculations.

### Base case scenario

In this analysis, the base case scenario relies on the following set of assumptions: a discount rate of three percent has been applied to economic capital costs, and each ITN provides 3 years of protection for two people and assumes 100% of nets distributed are being used. One-way sensitivity is then used to test these assumptions on the economic cost per person year of protection from the full provider perspective.

## Results

Table 1 provides a summary of key program characteristics of each of the programmes included in this costing analysis. The information includes: the type of distribution channel, the year in which the costed distribution took place, the number of regions, districts, and health facilities/schools served, whether the area served was rural, urban or a mixture, the total number of nets distributed, and the population targeted.

**Table 1:** Summary of key programme characteristics

Country	Channel	Year	Number of regions served	Number of districts	Number of health facilities (HF) or Schools	Area type	Number of nets distributed	Population target
Mali	Mass distribution	2015	1 region (Segou)	8 districts	198 health facilities	Rural	1,752,092	Entire population of Segou
Mali	Mass distribution	2015	1 region (Bamako)	6 districts	60 health facilities	Urban	1,571,834	Entire population of Bamako
Mali	Continuous: Health Facility (ANC + EPI)	2015	9 regions	68 districts	1287 health facilities	Rural, urban	992,267	Pregnant women, children under 5 completing first series of vaccines
Ghana	Continuous: Health Facility (ANC + EPI)	2015	5 regions	119 Districts	3750 health facilities	Mostly rural	584,700	Pregnant women, children under 5 completing first series of vaccines
Ghana	Continuous: Health Facility (ANC + EPI)	2016	9 regions	208 districts	6284 health facilities	Rural, urban	710,888	Pregnant women, children under 5 completing first series of vaccines
Ghana	Continuous: schools	2016	6 regions	150 districts	16,136 schools	Mostly rural	909,650	Primary school children
Tanzania (Mainland)	Continuous: schools	2015	3 regions	19 districts	1919 schools	Mostly rural	494,407	Primary school children
Tanzania (Mainland)	Continuous: Health Facility	2016	7 regions	46 districts	713 health facilities	Mostly rural	793,320	Pregnant women, children under 5 completing first series of vaccines
Tanzania (Zanzibar)	Continuous: Community-based Distribution and Health Facility (ANC + EPI)	2015	1 region	10 Districts	160 health facilities 327 *Shehias*	Mostly rural	216,310	Pregnant women, children attending epi, community members who meet eligibility requirements
Tanzania (Mainland)	Continuous: schools	2017	14 regions	105 districts	9535 schools	Mostly rural	3,041,139	Primary school children

ANC, antenatal care; EPI, Expanded Programme on Immunization; *Shehias,* Shehia is the lowest official administration unit in Zanzibar and each Shehia consists of a number of villages and households. Shehas (Shehia leaders) are tasked with distributing net vouchers to individuals who qualify for a net; vouchers are exchanged for nets at health facilities

### Mali intervention descriptions

#### Mass distributions

The mass distributions in Mali aimed to cover the entire population in Bamako (an urban setting) and Segou (a rural setting) by providing one ITN for every two persons. Before distributing the ITNs, advocacy and planning meetings were held with regions and districts, as well as trainings and microplanning. The population targeted for mass distribution was enumerated and registered. During the household registration, the heads of each household were provided vouchers that could be exchanged for a net at the time of distribution. The ITNs for the mass distributions were stored in several large warehouses in Bamako before being transferred to several health districts, in the case of the Segou mass distribution; or directly to the health facilities, in the case of the Bamako mass distribution. At all levels (districts and health areas) local government officials identified and paid for the storage warehouses and set up a system for securing the ITNs throughout the storage period before their distribution. Logistics plans were developed for the transportation, warehousing, and pre-positioning of the ITNs for all levels. Commercial haulers transported the ITNs via road to the health districts in Segou and Bamako. After the ITNs were delivered to the county seats of the selected districts—or directly to the health areas, in the case of Bamako—the district health authorities were responsible for pre-positioning the ITNs at the distribution points. Social and behaviour change communication (SBCC) plans were also developed and implemented. During distribution, community liaisons handled the distribution of the nets, in collaboration with community health associations. Heads of households travelled to specific distribution points to exchange their vouchers for nets. If more or fewer nets than were originally planned for were needed, additional personnel were sometimes hired to assist with the process of redirecting nets to meet requirements. Local civil society organizations were also encouraged to support some of the costs.

#### Continuous distribution: health facility (ANC + EPI)

The Ministry of Health (MoH) provided free nets to pregnant women at their first ANC visit and to infants when they complete their national immunization series. Nets were distributed to health facilities within all regions of Mali. Because this process has been ongoing for an extended period, little refresher training or additional planning was necessary. Nets are stocked year-round in central storage warehouses in Bamako. Transportation of the ITNs to the health districts from central storage took place via road, on a semi-annual basis, using commercial haulers selected by the implementing partner. After the ITNs were delivered to the districts, the district health authorities were responsible for allocating the ITNs to the health facilities, based on requisition forms that health facilities submitted monthly. Typically, health facilities store a 3-month supply of nets; however, this varied depending on the facilities’ storage capacity and was, therefore, inconsistent across health facilities. While other health facility distributions programs often suspend distributions during mass distribution years, Mali’s health facility distributions continued operating in Bamako and Segou. CD utilized human resources at the district and health facility levels throughout the year. These personnel assisted with the transport, storage, and issuing of ITNs; supervision, planning, training, and data reporting. Health facility personnel retrieved nets from district warehouses and transported them (with fuel costs typically reimbursed by the district) to health facilities for storage and they provided nets to patients along with counselling and advice on ITN use as part of the ANC/EPI visit.

### Ghana intervention descriptions

#### Continuous distribution: health facility (ANC + EPI)

For the CD of ITNs through health facilities in 2015–2016, nets were procured internationally, after which they were shipped to Ghana, cleared through customs and delivered to central medical stores (CMS). Before CD activities began, nets for all three channels of distribution (ANC, EPI, and schools) were delivered to either regional or district stores. The central/regional medical stores department typically distributed the nets with reimbursement for transportation costs, paid for with funds provided to the NMCP by international NGOs. Once nets arrived at the district or regional stores, they would then be delivered to health facilities based on demand forecasts. Occasionally, sub-districts helped facilitate this process (particularly in larger districts). They also provided additional storage space, assisted with the transport of nets to the health facilities and reporting. MoH personnel assisted with the transport, storage, and issuing of ITNs; supervision, planning, training, and data reporting. Health facility personnel retrieved nets from district warehouses and transported them (with fuel costs typically reimbursed by the district) to health facilities for storage and they provided nets to patients along with counselling and advice on ITN use as part of the ANC/EPI visit.

#### Continuous distribution: schools

For the school distribution in 2016, the nets were procured internationally, shipped to Ghana, cleared through customs and delivered to regional health stores, district governments, circuit government, or directly to schools. Once nets arrived at the district or regional stores, they were then delivered to schools based on assessment of past enrollment records that had been validated by School Health Education Programme (SHEP) officers. Nets distributed to schools are typically distributed *en masse* once per year to students enrolled in primary school classes two and six. Nets were distributed as soon as possible after delivery (often the next day) to avoid protracted storage at the school. Students were enumerated using concurrent enrollment registers or through the use of Education Ministry Information System data and nets were directly distributed by teachers. Implementing partners and the Ministry of Education supervised the school distribution through SHEP officers and school principals. Implementing partners also provided additional communication, health promotion, and behaviour change communication support, with assistance from other international NGOs. Implementing partners and the NMCP also provided training of trainers and support for regional and district-level training for net distribution activities.

### Tanzania intervention descriptions

#### Continuous distribution: health facility (ANC + EPI)

For the 2016 continuous health facility-based distribution channels in mainland Tanzania, ITNs were imported to Tanzania through a procurement agent, which contracted a local logistics firm to transport them from the port to one of four private warehouses. The process of managing nets from their arrival at the central warehouses through their transport to health facilities (*i.e.* health centres and dispensaries) was taken on by a number of international stakeholders and a logistics firm was sub-contracted to physically transport the ITNs. The project began with a “Smart Push” strategy in which each health facility received an initial supply of ITNs. The numbers that each facility received were planned based on patient volume data. Following the “Smart-Push,” facilities report quarterly on the number of nets they have distributed and are re-stocked accordingly. The nets are given free-of-charge to pregnant women at their first ANC visit, and to children at 9 months when they receive their first measles rubella vaccination.

#### Zanzibar continuous distribution: community-based and health facility (ANC + EPI)

The 2015 combined community-based and health facility CD in Zanzibar distributed the vast majority of ITNs through a community mechanism (61%) while the remainder of the nets were distributed through the ANC and EPI systems (20% and 19%, respectively). The community system functioned through a mechanism by which *shehia* (local political/community leaders) were empowered to issue coupons to any eligible resident of their area to receive an ITN. A resident could then present the coupon at a health facility (where all CD nets were stored) in exchange for an ITN. The system at the *shehia* for determining eligibility consisted of a committee working with the *sheha* to determine whether the applicant for an ITN met pre-specified eligibility criteria. Eligibility criteria were as follows: absence of an adequate number of ITNs in the home during reactive case detection activities, the resident is an orphan, resident is a widow/widower, resident is disabled, resident’s net was lost or destroyed in a disaster (such as flood or fire), the resident has recently moved/returned to the area, the resident’s ITN is damaged/severely worn, or the resident’s household does not have sufficient ITNs. Some coupons for ITNs were issued directly during reactive case detection by district malaria surveillance officers.

Besides those nets given through the community/*shehia* mechanism, all other nets in the CD system were distributed through health facilities. Eligible patients at facilities were issued coupons which can be redeemed at the same facilities in exchange for an ITN at no cost. Eligibility in this system was determined by the following criteria: for ANC, at a woman’s first ANC visit for each pregnancy, or for EPI, measles vaccination.

ITNs were distributed directly from the national level to the health facilities, where all CD nets were stored and all coupons for CD nets were redeemed. Districts and *shehias* play no role in transport or storage of ITN. Reporting on net distribution proceeded from *shehias* and health facilities through districts to the national level. Districts additionally played a role in supervision and training. SBCC and other communication activities could also be coordinated through districts, but all mass media activities were coordinated at the national level. Support for quantification in this system was provided by international consultants.

#### Continuous distribution: schools 2015

ITNs for the 2015 Tanzania school distribution were procured and delivered to a hired warehouse in Dar es Salaam. Before distributing the ITNs, advocacy and planning meetings were held with regions and districts, as well as trainings and microplanning. Students were enumerated by local education authorities using enrollment registers of the target classes and a validation exercise was conducted to ensure the accuracy of these numbers. After re-quantification was completed and verified, and before distribution activities began, nets were delivered to the districts and then transported to all schools in each district, based on the approved, validated quantifications for each school. At the school level, teachers were trained prior to ITN delivery. Nets were distributed as soon as possible after delivery, ideally the next day, to avoid prolonged storage at the school. ITNs were distributed to schoolchildren in primary school classes 1–3, 5, and 7 in Mtwara and Ruvuma and classes 1–5 and 7 in Lindi. Members from the national, regional, district, and ward levels formed supervision teams who oversaw the trainings, transportation, and distribution of ITNs in the selected districts. Additional behaviour change communication support was provided in the form of mass media and print materials.

#### Continuous distribution: schools 2017

SNP4 scaled up the programme from three regions in 2015 to seven regions in 2016 and SNP5 targeted 14 regions in 2017. The number of classes targeted varied by region in relation to previous distributions. Selection of classes was based on provisional enrollment data from a President’s Office Regional Administration and Local Government (PO-RALG) system (Basic Education Management Information System (BEMIS)) as of June 30, 2017, earlier NetCALC universal coverage modelling, and consultation with NMCP. Engagement and coordination meetings to involve key stakeholders in the implementation of SNP5 were conducted by international stakeholders and the NMCP at the national, regional and district levels. Trainings were also held in the seven regions that had implemented SNP in the previous round (SNP4), as well as the seven new regions.

Nets were provided by two international donor sources and procurement was handled differently for the different net sources. Nets from one source were manufactured in Arusha and transported to regional centres and then transported by a logistics agent directly to the schools for distribution. The remaining nets were manufactured internationally and shipped to Dar es Salaam. A clearing agent cleared them through customs, and the same logistics agent used for the other nets, transported the nets directly to the schools for distribution.

Prior to distribution international stakeholders and the logistics agent developed detailed microplans and desk validated ITN quantities. Prior to delivery to schools, the logistics agent used mobile phone applications to ensure that deliveries were made to the right locations in the right quantities. Original shipping containers were transported to a central point in each of the regions where nets were offloaded and delivered as soon as possible (usually the same or next day) to schools. This avoided the need for storage at regional level or at the schools. Supervision teams included representatives from the national levels from the NMCP and international stakeholders, regional and district-levels, with some involvement from the wards. A local marketing and promotion agent was contracted to conduct SBCC activities prior to and during distribution in selected regions.

### Costs of distribution programmes

#### Total financial and economic costs of distribution programmes

Total financial and economic costs and cost per net distributed, along with the total number of nets distributed for all programmes analysed are presented in Table 2. Overall, the total financial costs of the programmes ranged from 5,756,634.26 Int$ to 21,250,781.49 Int$ and 1,804,659.77 USD to 10,426,531.46 USD. Economic costs of the programmes ranged from 5,578,682.26 Int$ to 21,738,690.63 Int$, and 2,140,926.00 USD to 10,818,376.71 USD. The total financial and economic costs of the program varied substantially across all programmes in both USD and Int$. The total economic costs and financial costs of the programmes (USD) were generally a reflection of the number of nets distributed, however because of the price level differences between countries when converting from USD to Int$ this effect was reduced in the Int$.

**Table 2:** Total financial and economic costs of ITN Distribution systems and cost per net distributed

	Number of nets	Total cost of programme				Total cost per net distributed			
		USD (2016)		INT$ (2016)		USD (2016)		INT$ (2016)	
		Financial	Economic	Financial	Economic	Financial	Economic	Financial	Economic
Mali MD -SG 2015	1,752,092	7,333,350	7,651,944	10,543,031	10,868,670	4.19	4.37	6.02	6.20
Mali MD-BKO 2015	1,571,834	6,898,617	7,252,833	10,257,681	10,720,818	4.39	4.61	6.53	6.82
Mali CD-health facility 2015	992,267	8,159,764	8,420,389	15,987,483	16,372,390	8.22	8.49	16.11	16.50
Ghana CD-health facility 2015	584,700	4,378,316	4,670,309	7,935,128	8,482,691	7.49	7.99	13.57	14.51
Ghana CD-health facility 2016	710,888	5,685,228	6,103,374	10,995,841	11,846,269	8.00	8.59	15.47	16.66
Ghana CD- School 2016	909,650	3,689,828	3,907,755	5,756,634	6,091,325	4.07	4.31	6.35	6.72
Tanzania CD-School 2015	494,407	4,386,128	4,490,695	10,917,384	11,080,789	8.87	9.08	22.08	22.41
Tanzania CD-health facility 2016	793,320	5,950,946	6,302,993	14,949,635	15,827,027	7.50	7.95	18.84	19.95
Zanzibar CD-community + health facility 2016	216,310	1,804,660	2,140,926	4,546,788	5,578,682	8.34	9.90	21.02	25.79
Tanzania CD-school 2017	3,041,139	10,426,531	10,818,377	21,250,781	21,738,691	3.43	3.56	6.99	6.99

Total financial costs are represented in 2016 International Dollars and 2016 USD. Unit economic costs are represented in International Dollars and 2016 USD

#### Unit financial and economic costs of ITN Programmes

**Fig. 1:**
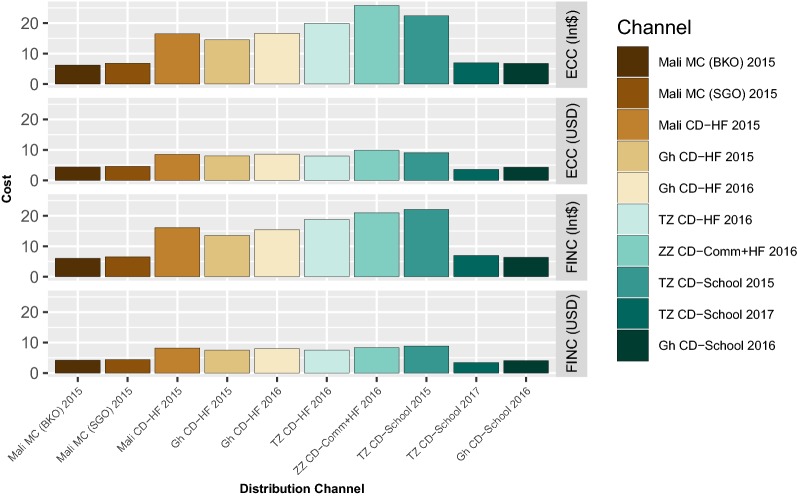
Unit Financial and economic costs of ITN distribution systems. Unit economic costs are represented in International Dollars (ECC INT$) (Panel 1) and 2016 USD (ECC USD) (Panel 2). Unit financial costs are represented in International Dollars (FINC INT$) (Panel 3) and 2016 USD (FINC USD) (Panel 4)

Unit financial and economic costs for all programmes analysed are presented in Fig. 1.

Overall the financial costs per net ranged from 6.02 to 22.08 Int$ and 3.43 USD to 8.87 USD. The financial costs per net (Int$) were generally much higher for nets delivered in Tanzania compared to those delivered in Ghana and Mali due to price level differences between countries. The financial costs of nets delivered through continuous systems via health facilities and schools were generally higher than those delivered through mass distributions, with Ghana’s school distribution and Tanzania’s 2017 school distribution as the exceptions. The financial costs per net delivered through the health facility-based continuous systems were relatively consistent for each of the programs analysed. Economic cost per net ranged from 6.20 Int$ to 25.79 Int$, and 3.56 USD to 9.90 USD. The economic costs per net (Int$) were generally higher for nets delivered in Tanzania (including Zanzibar) compared to those delivered in Ghana and Mali. The economic costs of nets (USD) delivered through continuous systems via health facilities and schools were, in general, higher than those delivered through mass distributions, with Ghana school distribution and Tanzania’s 2017 school distribution as the exceptions. The economic costs per net (USD) delivered through the health facility-based continuous systems were relatively consistent for each of the programmes analysed, with Zanzibar’s combined community + health facility programme incurring the highest cost.

**Fig. 2:**
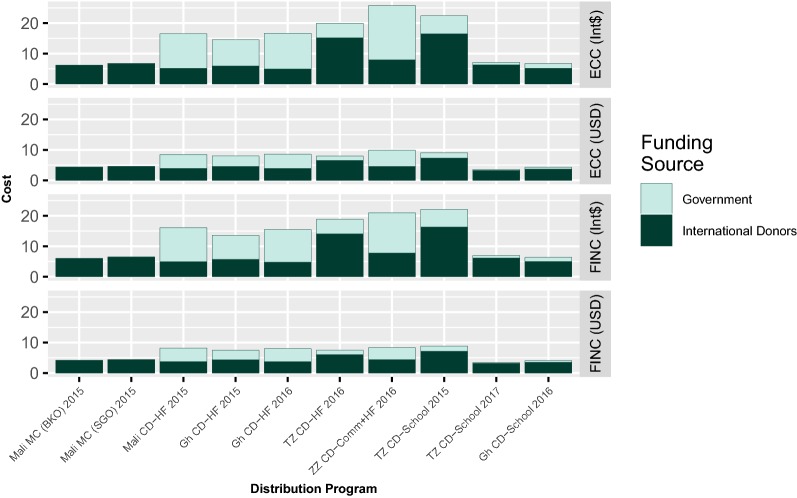
Unit financial and economic cost of distribution of ITN distribution systems (full provider perspective and international donor only). Economic Costs in International Dollars (ECC INT$) (Panel 1) and 2016 USD (ECC USD) (Panel 2). Financial costs in 2016 International Dollars (FINC INT$) (Panel 3) and 2016 USD (FINC USD) (Panel 4)

Figure 2 depicts the unit financial and economic cost of distribution (including the costs of the nets) from both the full provider perspective and the international donor perspective for all programmes analysed. The share of the total cost of the programmes paid for by the government was much higher when adjusted for price level. ITNs are classified as a tradable good, the Int$ costs of ITNs are the same as those reported in USD, while all other costs are higher. International donors paid the cost for all of the ITNs distributed, thus their relative contribution to the total cost of the programme is lower when adjusting for price level.

The proportion of costs provided by international donors was higher than that provided by the government for both mass distributions, Ghana’s school-based CD, Tanzania’s school-based distributions and Tanzania’s health facility-based CD in both the economic and financial analysis. The proportion of contribution from domestic sources was greater than 50% in Mali and Ghana’s health facility-based continuous distributions and Zanzibar’s combined community and health facility-based CD.

Overall, CD systems required substantially more investment from domestic sources compared to mass distributions. While international donors generally covered the costs of important line items such as nets, transportation, and central storage, the government provided financial and economic resources in the form of personnel, vehicles, storage space at local levels, as well as additional ITN transportation costs locally in many settings.

When only the international donor perspective is considered, the Tanzania mainland CD programmes incur the highest economic and financial costs per net, especially when adjusting for price level in the Int$ conversion.

#### Economic costs per person year of protection and per treated net year

Table 3 depicts the economic costs per person year of protection and per treated net year for all programmes analysed. The cost per person year of protection was based on the assumption that one net protects two people over a 3-year ITN lifespan, assuming that 100% of nets that are distributed are used. Similarly, the cost per treated net year assumes a 3-year ITN lifespan.

**Table 3:** Economic costs per person year of protection and per treated net year

	Economic cost per PYP (2016 USD)	Economic cost per TNY (2016 USD)
Mali MD (SG) 2015	$0.73	$1.46
Mali MD (BKO) 2015	$0.77	$1.54
Mali HF-CD 2015	$ 1.41	$2.83
Ghana HF-CD 2015	$ 1.33	$2.66
Ghana HF-CD 2016	$1.43	$2.86
Ghana school CD 2016	$ 0.72	$1.44
Tanzania school CD 2015	$1.51	$3.03
Tanzania HF-CD 2016	$1.32	$2.65
Zanzibar community + HF-CD 2016	$1.65	$3.30
Tanzania school CD 2017	$0.59	$1.19

Because the same base case scenario has been applied to all programmes, the relative economic cost per person year of protection and the relative economic cost per treated net year reflect same ordering as when the comparison is based solely on unit costs (per net distributed).

#### Line item breakdown of financial and economic costs of ITN programmes

**Fig. 3:**
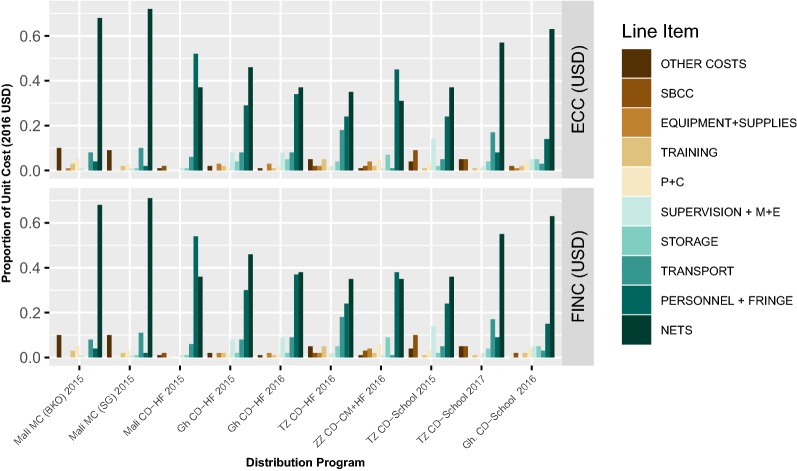
Line item breakdown of costs in ITN programmes. Economic costs (ECC) (Panel 1) and Financial Costs (FINC) (Panel 2) in 2016 USD

The line item breakdown of the financial and economic costs for all programmes analysed are presented in Fig. 3. The breakdown of line items is generally very similar in both the economic and financial analyses. The largest single line item for the two mass distributions in the economic analysis were ITNs. For the health facility-based CDs (including the Zanzibar combined community/HF-based distribution) the largest line items were personnel and fringe and ITNs, in the economic analysis. For school-based distributions, the largest line items were nets followed by personnel and fringe (Tanzania 2015 and Ghana 2016) or transport (Tanzania 2017), in the economic analysis.

The proportion of the total financial and economic costs made up by ITNs were generally highest in mass distributions, followed by the school net distribution in Ghana and the most recent school distribution in Tanzania. The other distribution systems had a similar proportion of costs attributed to ITNs. For the total costs made up of personnel and fringe (human resources) alone, the health facility-based distributions generally had the highest proportion of costs attributed to human resources. All the continuous systems, however, incurred substantial personnel and fringe costs, compared to mass distributions. The proportion of the total financial and economic costs made up by transport, were comparable for all distributions systems. However, the Zanzibar combined community/HF-based programme had the lowest proportion of costs attributed to transport in the economic analysis. The proportion of the total financial and economic costs made up by storage were also generally comparable for all distribution programmes, with Ghana school-based distribution and Tanzania HF-based distribution having the highest share of storage-attributed costs. The CD programmes generally had higher proportions of costs attributed to supervision and monitoring evaluation compared to the mass distributions. Tanzania’s 2015 school-based distribution had the highest proportion of costs attributed to supervision and M&E; this is likely due to the aforementioned re-quantification and validation exercises that were required after issues with enumeration. The proportion of costs made up by training activities were relatively consistent for all programmes, however, Mali’s HF-based distribution had the lowest share of costs attributed to training. Tanzania’s health facility-based distribution had the highest share of costs attributed to training, compared to the other programmes, likely due to the fact health facility distribution was completely overhauled and substantial changes were introduced. The proportion of the total financial and economic costs made up by planning and coordination costs were generally higher in mass distributions, while they were lowest in Mali’s health facility-based system. The proportion of the total costs made up by SBCC costs was substantially higher for Tanzania’s school-based distribution programmes compared to the other programmes.

### Sensitivity analysis

One-way sensitivity analysis testing base-case assumptions on the economic cost per person year of protection from the full provider perspective is shown in Table 4. The base case scenario assumes that one ITN provides 3 years of protection for two people and assumes 100% of nets distributed are being used.

**Table 4:** One-way sensitivity analysis of the cost per person year of protection

Distribution programme	Base case cost PYP	Country-specific use:access ratio	2 year lifespan	1.68 people per net	Fixed net price at 3.00 USD
Mali mass distribution (Segou) 2015	0.73	0.74	1.09	1.46	0.70
Mali mass distribution (Bamako) 2015	0.77	0.78	1.15	1.54	0.74
Mali continuous distribution: health facility-based (ANC + EPI) 2015	1.41	1.44	2.12	2.83	1.39
Ghana continuous distribution: health facility-based (ANC + EPI) 2015	1.33	2.05	2.00	2.66	1.22
Ghana Continuous Distribution: Health Facility-Based (ANC + EPI) 2016	1.43	2.20	2.15	2.86	1.40
Ghana continuous distribution: school-based 2016	0.72	1.11	1.08	1.44	0.77
Tanzania continuous distribution: school-based 2015	1.51	1.82	2.27	3.03	1.45
Tanzania continuous distribution: health facility-based (ANC + EPI) 2016	1.32	1.60	1.99	2.65	1.35
Zanzibar continuous distribution: community + health facility-based (ANC + EPI) 2016	1.65	1.99	2.48	3.30	1.64
Tanzania continuous distribution: school-based 2017	0.59	0.71	0.89	1.19	0.76

ITN use, obtained from population surveys, is defined as the proportion of the population that slept under an ITN the night before the survey. The use:access ratio gives an estimate of the proportion of the population using nets, among those that have access to one within their household. This indicator provides data on the behavioural gap for net use—rather than a gap because not enough nets are available [8]. The most recent use:access estimates from each country that costs were collected from were used to test the 100% use assumption. Use:access in Tanzania ranged from 0.69 to 1.05 across all regions in 2015 with an average use:access ratio of 0.83, use:access in Ghana ranged from 0.34 to 0.81 across all regions in 2014, with an average of 0.65 [47]. Between 2006 and 2010, the use:access ratio for almost all regions in Mali increased to above 0.80, and this was maintained through the 2015 survey with a range of 0.98–1.08 [47].

The base case analysis assumes that 100% of ITNs distributed are used. When the use:access assumption is adjusted to reflect actual use:access estimates from the countries in which these programmes were implemented (0.83 for Tanzania, 0.65 for Ghana, and 0.98 for Mali), substantial increases in the cost of the programmes per person year of protection were observed. A reordering of the cost per PYP was also observed. Tanzania’s 2017 school-based distributions remained the lowest cost per PYP, however the mass distributions replaced Ghana’s school-based distributions as the next lowest cost per PYP, followed by Ghana’s school-based distributions, followed by Mali’s health facility-based distribution, followed by Tanzania’s health facility-based distribution, followed by Tanzania’s 2015 school-based distribution, and the health facility-based distributions in Ghana replace Zanzibar’s combined community and health facility-based distributions as the most expensive programme per PYP. Use:access is, therefore, a very significant determinant in the efficiency of these programmes in the provision of PYP.

Net durability, or the useful life of an ITN, is assumed to be 3 years in the base case scenario. In reality the lifespan of ITNs may actually be closer to two rather than 3 years [30–33]. In this case, the impact of the intervention during year three of the ITN distribution-replacement cycle could be well below that seen in years one and two and would, therefore, have a significant effect on the cost effectiveness and efficiency of these programmes.

The base case analysis also assumes that two people sleep under a net each night. A study presenting survey data from several sub-Saharan African countries between 2008 and 2012 showed that the mean number of users per net is significantly lower when enough ITN were available (at least one net for every two people) (mean of 1.68 (95% CI 1.63, 1.71)) compared to if the household did not have enough ITN (mean 2.27 ITN users (95% CI 2.20, 2.36)) [48]. Therefore, to test the base case assumption, the number of users was adjusted to 1.68 users per net. This assumption is likely to vary from setting to setting due to different cultural and socioeconomic contexts, as well as differing levels of ITN ownership and is, therefore, also another important determinant for the efficiency of ITN distribution programmes.

The cost of the net itself makes up a substantial portion of the total cost of net programmes. This cost is also slightly variable from programme to programme, with a range of 2.02–3.64 USD per net, and has decreased substantially in recent years, therefore, the effect of using a constant net price is presented to remove potential variation in unit costs arising only from net purchase price. When a consistent net price is used, a reordering of the cost per PYP is observed. The mass distributions replace Ghana’s 2016 and Tanzania’s 2017 school-based distributions as the lowest cost per PYP, these school-based distributions drop to second lowest cost per PYP, followed by the health facility-based distributions, and then Tanzania’s 2015 school-based distributions, and then followed by combined community/HF distribution in TZ.

## Discussion

This study compared the cost of ITN distribution through mass campaigns, health facilities (ANC and EPI), schools, and community-based approaches. Costs for systems of these types were collected in three different countries. Overall, findings from the study showed that when the full provider perspective is considered, ITNs distributed through mass campaigns had a lower economic unit cost than those distributed through ANC/EPI-based systems. CD systems tend to distribute fewer nets compared to mass distributions, therefore their total costs are typically much lower than mass campaigns even though mass campaigns might have lower unit costs.

ITNs distributed through school-based CD also, generally, had a higher economic cost per net distributed compared to the mass distributions in Mali, when adjusted for price level, though the school-based net distributions in Ghana (2016) and in Tanzania (2017) had a slightly lower unit economic cost than these mass campaigns when reported in USD.

When the costs of domestic contributions are excluded, and donor financed components of the programmes are considered, the costs of the CD systems appear to be less costly than mass distributions. The 2015 school-based distribution and 2016 health facility-based distribution programmes in Tanzania were an exception. This was largely due to the fact that in Tanzania, distribution through these systems involved extensive international donor involvement at the time of the cost data collection, compared to the systems operating in Mali and Ghana. Health facility-based distribution in Tanzania was overhauled in 2016 and reintroduced in a new format and therefore required substantially more training, supervision, and M&E costs compared to the other programmes. The 2015 School Net Programme was also still very much in its pilot phase and, therefore, required substantially more initial investment and donor involvement, as well. Between 2015 and 2017, cost-cutting measures were introduced that resulted in the Tanzania’s school-based distribution becoming the least expensive programme per net distributed.

The costs of the two mass distributions per net distributed and person year protected are comparable to those in the school-based CD system in Ghana and, more recently, Tanzania. These costs reflect a full provider perspective including both international donor funded activities as well as in-country government contributions of staff and resources. Donors and other planners of ITN distribution systems need to consider in-country contributions in the planning process of identifying value for money or other measures of efficient ITN distribution systems. However, they need not necessarily consider in-country contributions as explicit costs from their budget perspective. Furthermore, many of the in-country contributions, while not strictly in-kind contributions, represent use of staff time and infrastructure that are pre-existing, non-fungible expenses. The realization of these elements as costs of ITN distribution occurs only because the Ministry has directed its resources (that have already been financially committed) to be used in the distribution of ITNs rather than in any alternative manner, or by utilizing spare capacity in the system to conduct these activities. When planning for future programmes, it is important to consider whether it is feasible for the government to direct their already paid for resources towards CD or whether implementing CD will overload already weak health systems with new activities. A clear example of this was the implementation of a community-based distribution programme in South Sudan. In this case, costs were substantially higher compared to similar programmes, because the programme was implemented in a context where government input, like vehicles, was not possible or was extremely limited [40].

The overall economic costs per net distributed through the CD systems were generally higher than that those of the mass distributions from a full provider perspective, with the exception of Ghana’s and Tanzania’s most recent school-based distribution. However, there are some key differences between the systems that may explain this. First of all, mass distributions tend to benefit from some returns to scale due to the fact that huge quantities of nets are distributed over a such short period of time, compared to routine systems that operate year-round and rely on health workers to issue ITNs to individuals visiting an ANC or EPI clinic or presenting a coupon from a community-based scheme. Additionally, due to the continuous nature of health-facility-based and community-based CD and the level of commitment and cooperation required from the government at all levels for this programme to function effectively, ANC and EPI distributions required more substantial domestic contribution. The government provided the largest share of resources in both the economic and financial analyses for nearly all of the health facility-based CD analysed, with the exception of those in Tanzania, where international donors took on additional cost responsibilities for which the Malian and Ghanaian governments had been responsible in their respective programmes. While international donors covered the costs of important line items such as nets, transportation, and central storage, the government provided financial and economic resources in the form of personnel, vehicles, storage space at the lower levels, and additional ITN transportation costs at the lower levels. While the school-based CD models analysed in this study were not purely continuous systems, but rather intermittent systems with annual delivery, they still required substantial cooperation and commitment from all levels of government, particularly the lower levels (districts/circuits and schools), to function effectively. The intermittent nature of this system is reflected in the generally lower cost to the government per net compared to ANC/EPI and community-based programmes (fewer human resources required for this system to operate compared to purely continuous systems seen in ANC/EPI/community-based).

When compared to studies that have previously attempted to ascertain the costs of CD programmes, the costs reported in this study are generally higher [5, 42, 43]. This is likely due to the difference in methodology. First of all, some costs presented in this study are reported in international dollars and, therefore, these costs appear much higher compared to those reported in US dollars or Euro. In the costing analysis conducted here, survey tools were developed to use at the lower levels to capture detailed information on time and resource use. When time and logistical constraints limited the ability to utilize surveys, detailed informant interviews were utilized to capture this information instead. In prior studies, programmatic records were often used to capture this type of information and often human resources at the lower levels may not have been considered as financial (or economic) costs to the programme unless they were explicitly budgeted for.

ANC and EPI distributions directly target groups with special biological vulnerabilities to malaria and might provide additional value per ITN compared to mass distributions targeting the entire population. Mass distributions are typically conducted every 3–5 years. Mass distributions provide high coverage and high equity, however, pregnancies and births that occur between mass distributions represent vulnerable populations potentially unprotected without effective CD programmes [49]. Additionally, campaign nets that degrade over time need to be replenished through CD channels to sustain high levels of coverage. Therefore, relying solely on campaigns without keeping up access through routine systems like ANC and EPI, would result in lower levels of population-wide ITN access [29]. This effect is even more apparent if the time between campaigns is assumed to 5 rather than 3 years, again, underlining the need for routine systems that provide constant access to ITNs [29].

The school channel delivered ITNs at a lower economic cost per net distributed, compared to ANC/EPI-based distribution systems. Pilot programmes in Tanzania have demonstrated that this system is a feasible method for rapidly and equitably distributing large quantities of ITNs [36, 37]. Initial results of the evaluated 2015 school net programme indicate that distributions through schools have successfully maintained coverage over a short time period, despite the Tanzania National Voucher Scheme (a health-facility-based ITN voucher programme) being discontinued [36]. Other pilot programmes have demonstrated that ITN distribution through schools and ANC provide complementary reach and can play an effective role in achieving and maintaining universal coverage [38]. Because the equity of this system is linked to the equity of school enrollment and attendance, the success of these programmes might vary depending on the setting. However, a pilot programme in Nigeria demonstrated ownership of school ITNs was nearly as equitable as for campaign ITNs and there was no significant oversupply or undersupply among households with ITNs [38].

Zanzibar’s combined community/health facility-based distribution had the highest unit cost, overall. However, this is not surprising, as other programmes have cited higher costs for this type of channel [40]. Pilot programmes of community-based ITN distributions have demonstrated improved ITN ownership, access and use following distributions, with some pilot programmes coming close to meeting universal coverage targets in the areas targeted [40, 41]. Longer periods of implementation should be further evaluated to determine whether community-based distribution can effectively maintain ITN coverage beyond the short term, and reach all wealth quintiles equitably [40].

The economic cost per PYP for all programmes were sensitive to net use, lifespan of the net, and the number of users per net. Use is an important determinant of the efficiency of these programmes. While there is substantial information available about how CD programmes affect net coverage and access, there is little information available on how the source of net affects net use. Understanding who uses nets distributed through CD programmes, and how often they are being used, will be critical for decision-making.

Surveys were used to obtain local level costs in the Ghana and Tanzania analyses (with the exception of the 2017 school-based distribution), however due to resource limitations and travel restrictions, a survey could not be used to obtain costs at the local levels in the Mali analysis and the 2017 Tanzania school-based distribution, therefore key informant interviews at local levels were used to make estimates for these costs. Thus, key differences in the in-country contributions (mostly incurred at the lower government levels) within these two settings may potentially be attributed to differences in methodology. To some extent the international donor analysis may mitigate this potential bias by removing most domestic resource contributions. In that sub-analysis the unit cost results appear largely comparable across countries.

While the survey instruments were useful for capturing resource use and expenditure information at the lower levels for the programmes in which they were used, the results at each unit observed varied. Because these units were not a representative sample, they could lead to biased estimates, and because there were only a small number of sampled units, it is not possible to extrapolate to the full programme with high levels of precision. Additionally, it is possible that recall or social desirability bias could lead to over- or under-reporting of resource use, which in turn, may result in biased or imprecise estimates of government contributions. In future cost evaluations, care to directly observe, measure, and estimate these parameters (particularly personnel time contribution) may help to measure government contributions to the delivery systems more precisely, as well as to estimate the overall financial and economic costs of the distribution systems more accurately.

This study also assumes that the costs associated with CD through health facilities at the lower levels were fixed rather than related to the number of nets distributed. Therefore, average costs were obtained using either the questionnaire data or informant interviews and applied to each district/sub-district/health facility/school to obtain an overall cost for each of the specified levels. Therefore, it is likely that the true cost of health facility-based distribution at these lower levels could differ from reported costs based on whether this assumption holds true. This could also have programmatic implications. When making this assumption, the overall costs of health facility-based CD seem to be driven by the number of health facilities, sub-districts, and health facilities served, as these levels require substantial resources (personnel, storage space, vehicles). However, it is important to reiterate that the major share of these local level costs is provided by local government rather than international donors.

It is also necessary to note that household costs (both direct and indirect) were excluded from this analysis. Therefore, the estimated cost of these programmes in terms of total societal resources is likely to be downwardly biased. However, it is unlikely that these household costs would have been substantial. Generally, ITNs were distributed within close proximity to the households (distribution points for mass campaigns, schools, health facilities, and *shehias*) therefore the travel time and travel-associated costs would have been minimal. Additionally, many of those costs would have been incurred anyway as children receiving nets through school distributions would have likely attended schools regardless and mothers receiving nets through ANC/EPI would have likely travelled to these clinics regardless for their antenatal care and their children’s immunizations. Furthermore, as nets were provided free of charge, households were not expected to make any contribution towards the cost of the net itself.

Another important consideration when comparing the costs between these different channels across the three settings is context. It is likely that various societal, political, environmental factors may introduce bias. This study presents selected case studies believed to be representative of different ITN programmes and delivery channels in sub-Saharan Africa. However, there may be significant differences in the performance and costs of implementation of these programmes in other settings. This may also explain the similarity between the costs of the two mass campaigns analysed. While these two campaigns were carried out in two different regions in Mali, one rural and one urban, the two campaigns were implemented in the same country within a similar time frame. It is likely that there would have been greater variability between the costs if the programmes analysed were implemented in two different countries. To address the effect of price level as a component of this, the international dollar was used. However, international dollars represent broad economy wide estimates of price level, and as such, differences may not be accurate for the specific elements contributing to price level differences in ITN programmes. Furthermore, the consideration of all delivery costs as non-traded goods and services in this study is likely to overestimate the fraction of non-traded goods and services in each programme, potentially biasing results towards systems which purchased fewer non-traded goods. Therefore, it is important to identify to what extent these country-specific factors may affect the cost of implementing these programmes in different settings.

Cost, even per net or user may be a poor indicator of cost-effectiveness as numerous additional factors could intervene to modify the ultimate health outcomes on which cost-effectiveness analysis is based. Ultimately decisions based on a measure of the efficiency of delivery, such as cost per person year of protection or per net distributed are a first step towards appropriate prioritization of resources but ultimately such decisions should be informed by well conducted cost-effectiveness analysis. For this reason, the data generated in this study, along with other information on the cost of ITN distribution from the literature should be incorporated into future cost-effectiveness studies which involve the accurate assessment of likely health outcomes from mass campaigns, CD and combined ITN distribution systems.

## Conclusions

This study shows, that when the full provider perspective is considered, school net programmes (with the exception of Tanzania’s 2015 round) and mass distributions had the lowest economic cost per net distributed (3.56–4.31 USD for school-based and 4.37–4.61 USD for mass distributions), compared to ANC/EPI (7.95–8.59 USD), Tanzania’s 2015 school-based distribution (9.08 USD), and combined community/HF programmes (9.90 USD). However, from the international donor perspective, there was little difference between the four channels studied (3.30–4.55 USD), with the exception of Tanzania’s 2015 school distribution (7.30 USD) and Tanzania’s 2016 health facility distribution (6.52 USD). Mass distributions are heavily financed by donors, while CD relies more heavily on domestic contributions. Due to the continuous nature of community and health facility-based CD and the level of commitment required from the government, community and health facility-based CD required more substantial domestic contribution. Donors and other planners of ITN distribution systems need to consider in-country contributions in the planning process, even though these may not have explicit budget implications. When planning for future programmes, it is important to consider whether it is feasible for the government to direct resources (that have already been paid for) towards CD, or whether implementing CD will overload already weak health systems with new activities. Additionally, it is important that countries are able to sustain investment in CD, and that the policy environment is amenable to the incorporation of CD into policy when piloted programmes prove successful. The results suggest that CD strategies can continue to provide nets for a similar unit cost to mass distributions from the perspective of the donor but will require additional (usually in-kind) domestic contributions (per net) compared to mass distributions. While CD may prove more costly than mass campaigns in some instances, government buy-in and investment in CD results in a sense of ownership of the programme and can lead to long-term sustainability under the right conditions.

## Data Availability

The datasets used and/or analysed during the current study are available from the corresponding author upon reasonable request.

## Abbreviations

ITNs: Insecticide-treated nets
WHO: World Health Organization
CD: Continuous distributions
ANC: Antenatal care
EPI: Expanded programme on immunization
NMCP: National Malaria Control Programme
SNP: School Net Programme
USD: United States Dollar
Int$: The International Dollar (Gheary- Kamis Dollar)
PYP: Person year of protection
TNY: Treated net year
SBCC: Social and behaviour change communication
MoH: Ministry of Health
CMS: Central Medical Stores
SHEP: School Health Education Program
ICP: Interpersonal Communication
PO-RALG: President’s Office Regional Administration and Local Government
BEMIS: Basic Education Management Information System
